# Comparison of different animal models for estimating genetic parameters for early growth traits and reproductive traits in Tianmu Sainuo sheep

**DOI:** 10.3389/fvets.2024.1349790

**Published:** 2024-05-16

**Authors:** Wenna Liu, Qingwei Lu, Sen Tang, Xue Pu, Yaqian Wang, Cuiling Wu, Xiangrong Hu, Wei Hong, Xuefeng Fu

**Affiliations:** ^1^College of Animal Science, Xinjiang Agricultural University, Urumqi, Xinjiang, China; ^2^Key Laboratory of Genetics Breeding and Reproduction of Xinjiang Wool-sheep & Cashmere-goat (XJYS1105), Institute of Animal Science, Xinjiang Academy of Animal Sciences, Urumqi, Xinjiang, China; ^3^Key Laboratory of Animal Genetic Breeding & Reproduction of Ministry of Agriculture, Xinjiang Academy of Animal Science, Urumqi, Xinjiang, China; ^4^Key Laboratory of Special Species Conservation and Regulatory Biology, Key Laboratory of Plant Stress Biology in Arid Land, College of Life Science, Xinjiang Normal University, Urumqi, Xinjiang, China; ^5^Zhejiang Sainuo Ecological Agriculture Company, Hangzhou, Lingan, China

**Keywords:** sheep, litter size, model evaluation, non-genetic factors, fixed effects, heritability

## Abstract

As the economic level of individuals rises, so too does the demand for mutton. Enhancing the breeds of mutton sheep not only boosts production efficiency and economic benefits but also fosters the sustainable growth of the mutton sheep breeding industry. Thus, this study examines the early growth and reproductive traits of Tianmu Sainuo sheep, analyzing the genetic interactions among these traits to furnish a theoretical foundation for refining breeding strategies and expediting the genetic advancement of this breed. The investigation compiled 29,966 data entries, involving 111 sires for birth weight (BWT) and 113 for other metrics. The data encompassed 10,415 BWT records from 1,633 dams, 12,753 weaning weight (WWT) records from 1,570 dams, 12,793 average daily gain (ADG) records from 1,597 dams, and 13,594 litter size (LS) records from 1,499 dams. Utilizing the GLM procedure in SAS 9.2 software, the study analyzed the non-genetic influences on lamb BWT, WWT, ADG, and LS. Concurrently, DMU software estimated the variance components across various animal models for each trait. Employing the Akaike Information Criterion (AIC) and likelihood ratio test (LRT), six models were tested, incorporating or excluding maternal inheritance and environmental impacts, to identify the optimal model for deriving genetic parameters. The findings reveal that birth year (BY), birth quarter (BQ), birth type (BT), age of mother (AM), and birth sex (BS) exerted significant impacts on BWT, WWT, and ADG (*p* < 0.01). Additionally, BQ and AM significantly influenced LS (*p* < 0.01). The most accurate genetic evaluation model determined the heritability of BWT, WWT, ADG, and LS to be 0.0695, 0.0849, 0.0777, and 0.1252, respectively.

## 1 Introduction

Since domestication, sheep have provided humans with meat, wool, skin, milk, and other by-products (1). With increasing global population and rising living standards, the demand for these products has increased. Currently, breeding meat-producing sheep has become a priority, with the development of high-yield breeds emerging as a critical challenge in the Chinese sheep industry. Tianmu Sainuo sheep, a new dual-purpose breed prized for its meat and skin, exhibit stable genetic traits, rapid growth, and superior meat quality.

In recent years, the study of sheep growth and reproductive traits has intensified, as these factors significantly influence farm profitability (2–7). Animal growth varies over time, displaying a pattern of steady increase, although growth rates differ among individuals. Enhancing key metrics such as birth weight (BWT), weaning weight (WWT), and average daily gain (ADG), and weight at identification can directly impact the economic value of mutton (8). Genetic parameters are crucial for assessing population characteristics and enhancing breeding programs; thus, establishing a suitable statistical model is essential for genetic parameter estimation. This model must account for individual direct genetic effects, maternal genetic effects, maternal permanent environmental effects, group effects, forage quality, and a range of fixed and random effects influencing genetic parameters (9–15). Consequently, selecting an appropriate animal model customized to the unique breeding conditions is imperative.

As Tianmu Sainuo sheep constitute a new herd, there is a lack of reports on the estimation of their genetic parameters for early growth and reproductive traits. Consequently, this study employed various animal models to estimate the variance components of these traits in Tianmu Sainuo sheep. This study aimed to identify the optimal model for each trait, enhance the precision of genetic parameter estimation, and analyze the genetic trends of the breed. This analysis will inform and refine future breeding programs for Tianmu Sainuo sheep, aiming to enhance their genetic quality and develop high-quality, high-yield breeds that align with societal needs, thereby providing a theoretical framework and reference for sheep breeding.

## 2 Materials and method

### 2.1 Data collection and description

This study’s data were sourced from Zhejiang Sainuo Ecological Agriculture, encompassing records from 2020 to 2022 on the growth and reproductive traits of Tianmu Sainuo sheep. In total, 29,966 records were gathered, detailing individual numbers, paternal and maternal individual numbers, birth sex (BS), birth date, birth type (BT), mother birth date, mother lambing days, mother lambing age, birth weight (BWT), weaning weight (WWT), litter size (LS), weaning date, and average daily gain (ADG). Table 1 presents the analyzed data. Notably, 111 sires contributed to the BWT data, while 113 were involved in the other traits, including 10,415 BWT records from 1,633 dams, 12,753 WWT records from 1,570 dams, 12,793 ADG records from 1,597 dams, and 13,594 LS records from 1,499 dams. The selection criteria for Tianmu Sainuo sheep data were based on data distribution and prior knowledge (16–21). The data were organized in Excel for analysis. To enhance data analysis accuracy, entries with indistinguishable ewe ages due to missing or supplementary numbers were removed, along with extreme and abnormal values. Following the removal of the outlier, the weight data across various developmental stages of Tianmu Sainuo sheep adhered to a normal distribution. The growth traits analyzed were BWT, WWT, and ADG, while LS was the examined reproductive trait, using the least-squares method of variance analysis (GLM).

**Table 1:** Characteristics of the data structure for early growth traits and reproductive traits of Tianmu Sainuo sheep.

Item	BWT, kg	WWT, kg	ADG, g	LS
No. of animals	12,159	14,436	14,503	15,206
No. of records	10,415	12,753	12,793	13,594
No. of sires	111	113	113	113
No. of dams	1,633	1,570	1,597	1,499
No. of records per sires	91.40	112.86	113.21	120.3
No. of records per dams	6.38	8.12	8.01	9.07
Mean	3.16	15.30	288.55	2.46
S.D	0.54	2.18	52.32	0.82
C.V (%)	16.63	13.27	21.31	32.49

BWT, birth weight; WWT, weaning weight; ADG, average daily gain; LS, litter size; C.V., coefficient of variation.

### 2.2 Level division of non-genetic factors

This study examines the impact of non-genetic factors, birth year (BY), birth quarter (BQ), birth sex (BS), birth type (BT), and age of mother (AM) on the early growth traits of Tianmu Sainuo sheep, based on the sheep farm’s production situation and data structure. The BY effect is categorized into three levels corresponding to each year from 2020 to 2022. The BQ effect is segmented into four seasons: spring (March–May), summer (June–August), autumn (September–November), and winter (December–February of the subsequent year), with each season representing a level. The BS effect is bifurcated into two levels based on gender. The BT effect on LS is classified into three categories: single or more than four lambs, twin lambs, and triplet lambs. AM is delineated into four age groups: 1 year, 2 years, 3 years, and > 4 years. Notably, for the reproductive trait of LS, BT is excluded from consideration.

### 2.3 Statistical models

In this research, the selection criteria for data were based on normality test outcomes and actual production conditions, ensuring stringent data quality control. Using the GLM procedure in SAS software (22), we performed least squares analysis on the traits, setting the threshold for significance in multiple comparisons at *p* < 0.05 to minimize the likelihood of type I errors.

The statistical model of early growth traits is as follows:


$$Y_{\mathrm{ijklmn}}=u+a_{i}+b_{j}+d_{k}+t_{l}+h_{m}+e_{\mathrm{ijklmn}}$$

Where $Y_{\mathrm{ijklmn}}$ is the observation of the trait; u is the overall mean; $a_{i}$ represents the i-level effect of BY; $b_{j}$ represents the j-level effect of BQ; $d_{k}$ represents the k-level effect of sex; $t_{l}$ represents the l-level effect of BT; $h_{m}$ represents the m-level effect AM; $e_{\mathrm{ijklmn}}$ represents the random residual effect.

The statistical model of reproductive traits is as follows:


$$Y_{\mathrm{ijkmn}}=u+a_{i}+b_{j}+d_{k}+h_{m}+e_{\mathrm{ijkmn}}$$

Where $Y_{\mathrm{ijkmn}}$ is the observed trait value; *u* is the overall mean; $a_{i}$ represents the i-level effect of BY; $b_{j}$ represents the j-level effect of BQ; $d_{k}$ represents the k-level effect of sex; $h_{m}$ represents the m-level effect of AM; $e_{\mathrm{ijkmn}}$ represents the random residual effect. *p*-values > 0.05 are deemed non-significant, *p*-values < 0.05 are considered significant, and *p*-values < 0.01 are highly significant.

### 2.4 Genetic parameter estimation model

The (co) variance components for each trait were estimated using the restricted maximum likelihood (REML) method via the DMU software (23). Six distinct animal models were applied to each trait, varying in their inclusion of individual direct genetic effects, maternal genetic effects, maternal permanent environmental effects, and the covariance between individual and maternal genetic effects. Each model achieved normal convergence, a crucial aspect of the genetic algorithm that underscores the algorithm’s applicability. The models are defined as follows:


$$\mathrm{Model}\;I:Y=X_{b}+Z_{1a}+e$$

$$\mathrm{Model}\;II:Y=X_{b}+Z_{1a}+Z_{3\mathrm{me}}+e$$

$$\mathrm{Model}\;III\;:Y=X_{b}+Z_{1a}+Z_{2\mathrm{mg}}+e\;COV\left(a,m\right)=0$$

$$\mathrm{Model}\;IV:Y=X_{b}+Z_{1a}+Z_{2\mathrm{mg}}+e\;COV\left(a,m\right)=A\sigma_{\mathrm{am}}$$

$$\mathrm{Model}\;V:Y=X_{b}+Z_{1a}+Z_{2\mathrm{mg}}+Z_{3\mathrm{me}}+e\;COV\left(a,m\right)=0$$

$$\mathrm{Model}\;VI:Y=X_{b}+Z_{1a}+Z_{2\mathrm{mg}}+Z_{3\mathrm{me}}+e\;COV\left(a,m\right)=A\sigma_{\mathrm{am}}$$

Where *Y* represents the vector of observations for each trait; *b*, *1a*, *2 mg*, *3me,* and *e* denote the vectors of fixed effects, individual direct genetic effects, maternal genetic effects, maternal permanent environmental effects, and residual effects, respectively. *X*, $Z_{1}$ , $Z_{2}\text{,}$ and $Z_{3}$ are the design matrices linking the fixed effects, individual direct genetic effects, maternal genetic effects, and maternal permanent environmental effects, respectively. A symbolizes the direct genetic relationship matrix, while $\sigma_{\mathrm{am}}$ indicates the covariance between individual direct genetic effects and maternal genetic effects.

### 2.5 Comparison of different models

To ascertain precise genetic parameter estimates for early growth and reproductive traits in Tianmu Sainuo sheep, identifying the most suitable model among the six developed animal models is crucial. The Akaike Information Criterion (AIC) was employed to assess the variance components estimated by these models, aiding in selecting the optimal model. Concurrently, the likelihood ratio test (LRT) facilitated the evaluation of the merits and demerits of each model (24).

The AIC is calculated as follows:


AIC=2k−2lnL

In this formula, *L* signifies the maximized likelihood function value for the model under consideration (25), and *k* represents the number of parameters to be estimated within the model (26). The AIC addresses the influence of parameter count on the model’s efficacy, balancing the model fit against the risk of overfitting. Therefore, the model with the lowest AIC value is preferred (27), as it is indicative of the most effective variance component estimation (28).

The LRT is computed using the following formula:


$$LR=-2\log\frac{L1}{L2}=\left[-2\log\left(L_{1}\right)\right]-\left[-2\log\left(L_{2}\right)\right]$$

In the equation, LR denotes the likelihood ratio, with L1 and L2 representing the maximum likelihood function values for Models 1 and 2, respectively. Here, Model 1 serves as a submodel of Model 2. Additionally, LR follows a chi-square distribution, where the degrees of freedom equal the difference in the number of parameters between Model 2 and Model 1. A significant test result indicates a meaningful impact of the additional parameter on the trait; otherwise, the effect is considered negligible (29).

We employed the Akaike Information Criterion (AIC) and likelihood ratio test (LRT) to determine the most appropriate animal model. As a result, our findings are robust, effectively minimizing the likelihood of type I errors in our analysis.

### 2.6 Calculation of genetic parameters

Drawing on previous studies (30), we used variance components derived from DMU software to compute individual heritability, maternal heritability, maternal permanent environmental heritability, and the equations for phenotypic variance.

## 3 Results

### 3.1 Descriptive statistical analysis

The BWT, WWT, ADG, and LS values were 10,415, 12,753, 12,793, and 13,594, respectively. Their coefficients of variation were 16.63, 13.27, 21.31, and 32.49%, indicating a moderate level of phenotypic variability (10–60%) as shown in Table 1.

### 3.2 Least-square analysis of variance of early growth traits and reproductive traits

As presented in Table 2, the final fixed effects for each trait were determined. It was found that BY, BQ, BS, BT, and AM significantly influenced BWT, WWT, and ADG in Tianmu Sainuo sheep (*p* < 0.01). Specifically, BQ and AM had a significant impact on LS (*p* < 0.01). However, BY and BS showed no significant effect on LS (*p* > 0.05) (Table 3).

**Table 2:** The fixed effects used in different models for each trait.

Traits	BY	BS	BT	BQ	AM
BWT	√	√	√	√	√
WWT	√	√	√	√	√
ADG	√	√	√	√	√
LS				√	√

BWT, birth weight; WWT, weaning weight; ADG, average daily gain; LS, Litter size; BY, birth year; BS, birth sex; BT, birth type; BQ, birth quarter; AM, age of mother.

**Table 3:** Least-squares means ± S.D. for the studied traits.

Component	BWT, kg		WWT, kg		ADG, g		LS	
	*n*	$\bar{x}$	*n*	$\bar{x}$	*n*	$\bar{x}$	*n*	$\bar{x}$
BS		**		**		**		ns
Male	4,862	3.19 ± 0.01	6,264	15.94 ± 0.03	6,398	241.85 ± 0.64	6,817	2.47 ± 0.01
Female	5,553	3.08 ± 0.01	6,489	15.01 ± 0.03	6,395	212.93 ± 0.64	6,777	2.49 ± 0.01
BY		**		**		**		ns
2020	4,380	3.20 ± 0.01^a^	5,755	14.78 ± 0.03^c^	5,329	240.73 ± 0.70^a^	6,005	2.47 ± 0.01^a^
2021	2,678	3.09 ± 0.01^c^	3,068	15.66 ± 0.04^b^	3,304	216.15 ± 0.87^c^	3,396	2.50 ± 0.01^a^
2022	3,357	3.13 ± 0.01^b^	3,930	16.0 ± 0.03^a^	4,160	225.30 ± 0.79^b^	4,193	2.47 ± 0.01^a^
BQ		**		**		**		**
March to May	3,273	3.15 ± 0.01^b^	3,972	15.18 ± 0.03^c^	3,771	231.27 ± 0.82^a^	4,239	2.55 ± 0.01^a^
June to August	2,574	3.04 ± 0.01^c^	2,875	15.49 ± 0.04^b^	2,995	227.74 ± 0.91^b^	3,108	2.35 ± 0.01^c^
September to November	2,468	3.12 ± 0.01^b^	3,071	15.75 ± 0.04^a^	3,170	226.68 ± 0.91^b^	3,274	2.47 ± 0.01^b^
December to February of the next year	2,100	3.23 ± 0.01^a^	2,835	15.48 ± 0.04^b^	2,857	223.88 ± 0.94^c^	2,973	2.56 ± 0.01^a^
AM		**		**		**		**
1	3,373	3.05 ± 0.01^b^	3,921	15.17 ± 0.04^b^	3,849	218.17 ± 0.84^c^	4,107	2.26 ± 0.01^d^
2	3,008	3.18 ± 0.01^a^	3,758	15.63 ± 0.03^a^	3,837	231.95 ± 0.81^a^	4,026	2.50 ± 0.01^c^
3	1973	3.16 ± 0.01^a^	2,420	15.52 ± 0.04^a^	2,460	231.05 ± 1.00^a^	2,637	2.62 ± 0.02^a^
≥4	2061	3.16 ± 0.01^a^	2,654	15.59 ± 0.04^a^	2,647	228.40 ± 0.96^b^	2,824	2.55 ± 0.02^b^
BT		**		**		**		
Single or more than four lambs	1726	3.06 ± 0.01^c^	2,321	15.65 ± 0.04^a^	2,415	232.46 ± 1.00^a^		-
Twin lambs	4,547	3.26 ± 0.01^a^	5,783	15.64 ± 0.03^a^	5,788	230.32 ± 0.67^a^		-
Three lambs	4,142	3.09 ± 0.01^b^	4,649	15.15 ± 0.03^b^	4,590	219.40 ± 0.73^b^		-

The means with different letters in each sub-class within a column differ significantly from another. ** *p* < 0.01, * *p* < 0.05.

### 3.3 Comparison of different animal models

The analysis revealed that Model 1 produced the highest estimates of additive genetic variance and heritability for all traits. However, when incorporating maternal genetic effects, maternal permanent environmental effects, or the covariance between direct-mother additive genetic effects in subsequent models, there was a decrease in the estimates for individual direct genetic effect variance and heritability. The AIC demonstrated that Model 2 outperformed others in estimating genetic parameters for BWT and ADG before weaning, as shown in Table 4. This finding underscores the significance of the individual direct genetic effect and maternal permanent environmental effect in influencing Tianmu Sainuo sheep’s BWT and ADG. Conversely, Model 6 was most effective for WWT and LS genetic parameter estimation, highlighting the impact of individual direct genetic effects, maternal genetic effects, maternal permanent environmental effects, and their interactions on these traits. Furthermore, the LRT comparing the six models indicated no significant differences between Model 2 and Model 5 for BWT, ADG, WWT, and LS (*p* > 0.05). Similarly, no significant differences were found between Model 2 and Model 6 and between Model 3 and Model 4 for BWT and ADG (*p* > 0.05). Conversely, significant differences were observed between other model comparisons (*p* < 0.05).

**Table 4:** Standard values of AIC information for traits in different animal models.

Model	BWT, kg		WWT, kg		ADG, g		LS	
	−2logL	AIC	−2logL	AIC	−2logL	AIC	−2logL	AIC
1	−3106.96	−3102.96	30694.43	30698.43	112041.60	112045.60	6526.25	6530.25
2	−3234.45	−3228.45	30603.51	30609.51	111956.50	111962.50	5364.18	5370.18
3	−3180.74	−3174.74	30635.95	30641.95	111976.43	111982.43	5636.93	5642.93
4	−3183.29	−3175.29	30629.82	30637.82	111974.98	111982.98	5609.67	5617.67
5	−3234.67	−3226.67	30601.96	30609.96	111955.68	111963.68	5363.99	5371.99
6	−3235.54	−3225.54	30594.92	30604.92	111955.35	111965.35	5356.57	5366.57

−2logL, log-likelihood function; AIC, Akaike Information Criterio.

### 3.4 Estimation of variance components of early growth traits and reproductive traits using different animal model

As illustrated in Table 5, the heritability estimates of BWT across models varied, with Model 3 yielding the lowest at 0.0569 and Model 1 yielding the highest at 0.2775. Maternal heritability spanned from 0.0047 in Model 5 to 0.1566 in Model 4. The variance ratio attributable to maternal permanent environmental effects ranged from 0.1253 in Model 5 to 0.1292 in Model 2. WWT heritability estimates fluctuated between 0.0733 in Model 5 and approximately 0.2081 in Model 1. Maternal heritability for WWT was noted between 0.0096 in Model 5 and approximately 0.1162 in Model 4, with the variance ratio for maternal permanent environmental effects spanning from 0.0733 in Model 5 to approximately 0.0835 in Model 6. The heritability of ADG was estimated to be between 0.0632 in Model 3 and 0.1888 in Model 1, with maternal heritability ranging from 0.0133 in Model 5 to 0.1029 in Model 4. The variance ratio due to maternal permanent environmental effects was noted from 0.0685 in model 5 to 0.0795 in Model 2. For LS, heritability estimates varied from 0.0740 in Model 3 to approximately 0.5071 in Model 1. The variance ratio comparing maternal heritability to maternal permanent environmental effects was relatively low, ranging from 0.0042 in Model 5 to 0.4256 in Model 4, with additional figures of 0.3103 in Model 6 and approximately 0.3481 in Model 5.

**Table 5:** Variance components estimation by different animal models.

Traits	Model	$\sigma_{a}^{2}$	$\sigma_{m}^{2}$	$\sigma_{c}^{2}$	$\sigma_{e}^{2}$	$\sigma_{p}^{2}$	$h_{a}^{2}$	$h_{m}^{2}$	$h_{c}^{2}$	$\sigma_{\mathrm{am}}$
BWT, kg	I	0.0792			0.2061	0.2853	0.2775			
	II	0.0194		0.0360	0.2232	0.2785	0.0695		0.1292	
	III	0.0160	0.0336		0.2311	0.2807	0.0569	0.1196		
	IV	0.0224	0.0463		0.2268	0.2954	0.0757	0.1566		1.81E-05
	V	0.0187	0.0013	0.0349	0.2236	0.2785	0.0672	0.0047	0.1253	
	VI	0.0229	0.0035	0.0363	0.2209	0.2837	0.0808	0.0124	0.1279	5.60E-06
WWT, kg	I	0.8791			3.3452	4.2243	0.2081			
	II	0.3153		0.3380	3.4975	4.1509	0.0760		0.0814	
	III	0.3180	0.3099		3.5520	4.1799	0.0761	0.0742		
	IV	0.3822	0.5120		3.5083	4.4051	0.0868	0.1162		2.61E-03
	V	0.3044	0.0398	0.3042	3.5030	4.1514	0.0733	0.0096	0.0733	
	VI	0.3659	0.1175	0.3600	3.4648	4.3100	0.0849	0.0273	0.0835	1.73E-03
ADG, g	I	457.3046			1964.9238	2422.2284	0.1888			
	II	186.0535		190.1924	2017.0579	2393.3038	0.0777		0.0795	
	III	152.0285	192.8375		2062.0345	2406.9005	0.0632	0.0801		
	IV	168.5526	254.4218		2049.8634	2472.8378	0.0682	0.1029		4.25E+02
	V	172.9184	31.7447	164.0460	2024.7110	2393.4201	0.0722	0.0133	0.0685	
	VI	180.1883	47.2314	167.3471	2020.2034	2414.9702	0.0746	0.0196	0.0693	−4.07E+00
LS	I	0.3444			0.3347	0.6791	0.5071			
	II	0.0651		0.2083	0.3829	0.6563	0.0992		0.3174	
	III	0.0534	0.2685		0.4002	0.7222	0.0740	0.3718		
	IV	0.1027	0.3507		0.3704	0.8241	0.1247	0.4256		1.71E-04
	V	0.0648	0.0025	0.2059	0.3831	0.5915	0.1095	0.0042	0.3481	
	VI	0.0866	0.0204	0.2145	0.3698	0.6914	0.1252	0.0296	0.3103	7.94E-05

$\sigma_{a}^{2}$ , direct genetic variance; $\sigma_{m}^{2}$ , mother genetic variance; $\sigma_{e}^{2}$ , residual variance; $\sigma_{c}^{2}$ , mother permanence environmental variance; $\sigma_{\mathrm{am}}$ , direct and mother genetic covariance; $\sigma_{p}^{2}$ , phenotypic variance; $h_{a}^{2}$ , direct genetic effect; $h_{m}^{2}$ , mother genetic effect; $h_{c}^{2}$ , mother permanence environmental effect; “-”, without this effect in the model.

## 4 Discussion

### 4.1 The influence of fixed effects on early growth traits and reproductive traits

BY, BT, AM, BS, and LS exhibited significant impacts on BWT, WWT, and ADG (30–37). This could be attributed to variations in breeding management practices and climate changes across different years (38). Seasonal changes influence the nutritional quality of forage, thereby affecting lamb weights at various stages (39–41). Moreover, these factors also had a highly significant effect on LS (42). The notable influence of AM on early growth and reproductive traits might stem from behavioral and physical differences in ewes of varying ages (43, 44). Consequently, for precise genetic parameter estimation and effective breeding strategy development, a comprehensive consideration of non-genetic factors such as BY, BT, AM, and BS is essential.

### 4.2 Comparison of different animal models

Currently, animal models (45) are extensively utilized to leverage information from relatives, aiming to derive the most precise genetic parameter estimates. In this study, in addition to the individual direct genetic effect, random effects were incorporated to account for maternal genetic effects and maternal permanent environmental effects. In Model 1, where maternal genetic effects or both maternal genetic and permanent environmental effects are excluded, there is an observed increase in the estimated values for individual direct heritability (Table 5). Omitting maternal permanent environmental effects causes the total variation to be ascribed solely to maternal genetic variation, which may result in an inflated estimation of maternal heritability. For instance, in Model 5, compared to Model 3, estimates for maternal genetic variance rose from 0.0013 to 0.0336, and for heritability, the estimates rose from 0.0047 to 0.11196, respectively. Hence, both individual and maternal heritabilities are significantly influenced by the inclusion of random effects in the model. This pattern is consistent across other traits similar to BWT. Including maternal genetic effects, or both maternal genetic and permanent environmental effects in the model, leads to a reduction in individual genetic variance, maternal genetic variance, and their associated heritabilities.

For BWT and ADG, Model 2, which includes the individual direct genetic effect and maternal permanent environmental effect, demonstrated a lower AIC value compared to other models, indicating its superiority in accurately estimating these traits. The LRT results revealed that the differences between BWT and ADG were highly significant (*p* < 0.01), except for comparisons between Model 2 and Model 5, Model 2 and Model 6, Model 3 and Model 4, and Model 5 and Model 6, which were not significant (*p* > 0.05). This underscores the significant role of the individual direct genetic effect and maternal permanent environmental effect in genetic parameter estimation for BWT and ADG. For WWT and LS, Model 6, accounting for individual direct genetic effects, maternal genetic effects, maternal permanent environmental effects, and their interactions, exhibited a lower AIC value, indicating its effectiveness over other models. Notably, there were no significant differences in WWT between Model 2 and Model 5 (*p* > 0.05), yet significant differences were observed between Model 2 and Model 6, and Model 3 and Model 4 (*p* < 0.05), with other model comparisons showing highly significant differences (*p* < 0.01). Regarding the number of lambs born at the same birth, no significant difference was observed between Model 2 and Model 5 (*p* > 0.05), whereas significant differences were noted between Model 2 and Model 6 (*p* < 0.05). The differences between other models were highly significant (*p* < 0.01). These findings underscore the substantial impact of incorporating the interaction effect between the individual direct genetic effect, maternal genetic effects, and maternal permanent environmental effect on the genetic parameter estimation for WWT and LS. The results emphasize the critical role of considering various factors, including individual direct genetic effects, maternal genetic effects, and maternal permanent environmental effects, in the genetic parameter estimation for early growth traits, particularly WWT. Previous research has established the importance of maternal genetic effects in the accurate estimation of genetic parameters for these traits. For example, Maniatis et al. (46) illustrated that excluding maternal genetic effects from the model resulted in overestimated genetic parameter values for early growth traits. Tamioso et al. (47) highlighted the significant influence of maternal effects on early growth traits in lambs, such as BWT, WWT, and weight at 180 days. Tian’s (48) comparison of different animal models revealed the significant role of both individual direct genetic effects and maternal genetic effects in determining the BWT and WWT of Qinghai semi-fine wool sheep. Li et al. (49) identified a model that includes maternal genetic effects and maternal permanent environmental effects as most suitable for estimating genetic parameters for BWT and WWT in Aohan fine wool sheep. Studies by Gowane et al. (26) and Hanford et al. (50), through model comparisons, affirmed that BWT and WWT are significantly influenced by maternal genetic effects, aligning with the present study’s findings. Safari et al. (51), in their analysis of LS heritability in Australian Merino sheep, highlighted the necessity of incorporating individual direct genetic effects, maternal genetic effects, animal permanent environmental effects, maternal permanent environmental effects, and fetal effects in animal models. Hanford et al.’s (52) estimation of the heritability of sheep reproductive traits, which considered individual direct genetic effects and animal permanent environmental effects as random effects, supports some conclusions of this study. Therefore, careful selection of the statistical model is imperative for enhancing the accuracy of genetic parameter estimation for each trait.

### 4.3 Heritability analysis of early growth traits and reproductive traits

Heritability estimation is crucial for examining the genetic structure of traits (53). Heritability represents the proportion of phenotypic variance in a trait that is attributable to genetic variance within a population in a given environment. It not only reflects the capability of parents to transmit traits to their offspring but also informs future breeding strategies. The heritability of BWT in Tianmu Sainuo sheep was 0.0695, aligning with the findings in D’man (36), Barki (54), Moghani (55), and Lori sheep (56), yet it was lower than that observed in Sardi (57), Central Anatolian Merino (15), Shal sheep (58), Australian cotton (59), and Muzaffarnagari sheep (60). The relatively low heritability of BWT in Tianmu Sainuo sheep may be attributed to genetic modifications resulting from sustained breeding enhancements.

The heritability of WWT in Tianmu Sainuo sheep, recorded at 0.0849, matched the results for Alpine Merino (61) and Columbia sheep (52) but was lower than that reported for Baluchi (62), Chinese superfine Merino (63), Horro (64), and Australian Merino sheep (51). Variations in breeds, estimation models, and methods might explain these discrepancies.

For ADG, the heritability was 0.0777, consistent with Baluchi (62) and Moghani sheep (55) but lower than Marwari (65) and Harnali sheep (66). It is hypothesized that factors such as feeding management and artificial supplementation at birth could influence the heritability of WWT and ADG, potentially accounting for their lower heritability estimates.

The direct heritability of LS was 0.1252, aligning with the findings in Lori-Bakhtiari (67) and Chinese Merino (Xinjiang type) sheep (68). This heritability was higher than that reported for Merino (2), Arabi (69), and Ghezel sheep (70) but lower than in Baluchi (37) and Mehraban sheep (71). The relatively low heritability of LS in Tianmu Sainuo sheep may be attributed to the breed’s dual focus on meat and skin, with a predominant emphasis on meat traits, which could explain why LS heritability in this study is lower compared to other studies.

In summary, the heritability of early growth and reproductive traits varies among breeds, primarily due to differences in breeding programs, leading to genetic diversity within the same trait across breeds. This variation is partly due to the selection of fixed and random effects in the statistical analysis model and partly due to the unique characteristics of Tianmu Sainuo sheep and the data analyzed. Despite using data from 13,594 Tianmu Sainuo sheep and accounting for multiple non-genetic factors, the absence of some production and pedigree records may introduce a deviation between the estimated and actual genetic parameters. Furthermore, heritability is not only an intrinsic trait characteristic but also deeply intertwined with the environmental and feeding conditions of the subjects, which can significantly influence heritability estimates. It is crucial to acknowledge these limitations and contextual factors when interpreting this study’s results. Further research and analysis are essential to deepen the understanding of genetic parameters and heritability of traits in Tianmu Sainuo sheep.

## 5 Conclusion

This study represents the first examination of Tianmu Sainuo sheep, utilizing six distinct animal models to estimate the genetic parameters of their early growth and reproductive traits, subsequently identifying the most appropriate model for each trait category. Model 2 was found to be suitable for estimating the genetic parameters of BWT and ADG, while Model 6 was optimal for WWT and LS. The derived heritability estimates from the selected models revealed that BWT has a heritability of 0.0695, WWT has a heritability of 0.0849, and ADG has a heritability of 0.0777, categorizing these traits as having low heritability. In contrast, the heritability of LS was determined to be 0.1252, indicating moderate heritability. These findings lay a theoretical foundation for understanding the early growth and reproductive characteristics of Tianmu Sainuo sheep. They highlight the potential benefits of strategically eliminating ewes older than 6 years to optimize the genetic composition of the population. Moreover, the selection and retention of lambs with higher BWT during the initial rearing phase could markedly enhance the flock’s production performance. Such strategies are pivotal for improving the quality and productivity of the sheep population. In practice, it is essential to refine breeding programs continually based on genetic parameters, aiming to expedite the attainment of slaughter weight, thereby minimizing feeding costs and elevating breeding efficiency.

## Data availability statement

The original contributions presented in the study are included in the article/supplementary material, further inquiries can be directed to the corresponding authors.

## Ethics statement

The animal studies were approved by Animal Care and Use Committee of Institute of Animal Science, Xinjiang Academy of Animal Sciences. The studies were conducted in accordance with the local legislation and institutional requirements. Written informed consent was obtained from the owners for the participation of their animals in this study (Approval No. 2020001).

## Author contributions

LW: Conceptualization, Investigation, Methodology, Writing – original draft. LQ: Conceptualization, Investigation, Methodology, Writing – review & editing. TS: Software, Writing – original draft. PX: Investigation, Validation, Writing – original draft. WY: Formal analysis, Investigation, Writing – original draft. WC: Validation, Visualization, Writing – review & editing. HX: Data curation, Writing – review & editing. HW: Resources, Writing – review & editing. FX: Funding acquisition, Project administration, Writing – review & editing.
